# Dementia and hearing-aid use: a two-way street

**DOI:** 10.1093/ageing/afac266

**Published:** 2022-12-19

**Authors:** Graham Naylor, Lauren Dillard, Martin Orrell, Blossom C M Stephan, Oliver Zobay, Gabrielle H Saunders

**Affiliations:** School of Medicine, University of Nottingham, Nottingham, UK; Department of Otolaryngology- Head & Neck Surgery, Medical University of South Carolina, Charleston, SC, USA; VA Rehabilitation R&D, National Center for Rehabilitative Auditory Research, Portland, OR, USA; School of Medicine, University of Nottingham, Nottingham, UK; School of Medicine, University of Nottingham, Nottingham, UK; School of Medicine, University of Nottingham, Nottingham, UK; VA Rehabilitation R&D, National Center for Rehabilitative Auditory Research, Portland, OR, USA; VA Rehabilitation R&D, National Center for Rehabilitative Auditory Research, Portland, OR, USA; Manchester Centre for Audiology and Deafness, School of Health Sciences, University of Manchester, Manchester, UK

**Keywords:** hearing aid, dementia, causality, persistence, older people

## Abstract

**Objectives:**

Hearing-aid use may reduce risk of dementia, but cognitive impairment makes use more challenging. An observed association between reduced hearing-aid use and incident dementia could reflect either or both of these causal paths. The objective was to examine the effects of each path while minimising contamination between paths.

**Methods:**

Health records data from 380,794 Veterans who obtained hearing aids from the US Veterans Affairs healthcare system were analysed. Analysis 1 (*n* = 72,180) used multivariable logistic regression to model the likelihood of incident dementia 3.5–5 years post hearing-aid fitting for patients free of dementia and mild cognitive impairment (MCI). Analysis 2 (*n* = 272,748) modelled the likelihood of being a persistent hearing-aid user at 3 years 2 months after fitting, contrasting subgroups by level of cognitive function at the time of fitting. Analysis time windows were optimized relative to dataset constraints. Models were controlled for available relevant predictors.

**Results:**

The adjusted OR for incident dementia was 0.73 (95% CI 0.66–0.81) for persistent (versus non-persistent) hearing-aid users. The adjusted OR for hearing-aid use persistence was 0.46 (95% CI 0.43–0.48) in those with pre-existing dementia (versus those remaining free of MCI and dementia).

**Conclusion:**

Substantial independent associations are observed in both directions, suggesting that hearing-aid use decreases risk of dementia and that better cognitive function predisposes towards persistent use. Research studying protective effects of hearing-aid use against dementia needs to account for cognitive status. Clinically, hearing devices and hearing care processes must be accessible and usable for all, regardless of their cognitive status.

## Key Points

- Hearing-aid use shows a protective effect against dementia, but cognitive impairment hastens device disuse.
- Research studies targeting a protective effect must account for both pathways.
- To achieve a protective effect, hearing aids need to be usable by people with cognitive impairment.

## Introduction

Modifiable risk factors for dementia include untreated mid-life hearing loss [1], and it has been estimated that 8% of dementia cases globally are attributable to this factor [2]. Proposed mechanisms underlying the relationship between hearing loss and the development of dementia include (i) common underlying pathology (probably vascular), (ii) impoverished input affecting brain structure and function, (iii) cognitive resources overoccupied in listening unavailable for higher functions and (iv) interaction between auditory function and dementia pathology [3]. These mechanisms are not mutually exclusive.

The primary treatment for hearing loss is use of hearing aids. One may hypothesise that treatment with hearing aids will alleviate or decelerate cognitive decline (forward causal path: hearing-aid use → cognitive function), by counteracting one or more of the above mechanisms.

Meanwhile, since cognitive impairment is associated with difficulty in maintaining instrumental activities of daily living [4], it is also logical to hypothesise that adherence to recommended hearing-aid treatment is lower in those with cognitive impairment (reverse causal path: cognitive function → hearing-aid use). Ageing is strongly associated with sensory and cognitive decline [5] as well as increased risk of dementia. Thus, if hearing aids are to slow age-related cognitive decline, they must be used and usable by people whose cognitive function may already be impaired [6]. For this reason, and because both the forward and reverse causal paths will render negative associations (cross-sectional and longitudinal) between hearing-aid use and dementia, it is important to disentangle the effects of each hypothesised path.

Both longitudinal and cross-sectional quantitative studies have sought to test the existence of the forward path [7–16], with results in all except one [7] suggestive of an affirmative answer. However, while some of these studies [7, 8, 11, 13–15] refrain from attributing causality to observed associations, none attempt to control for the effect of the reverse causal path, and most only assess hearing-aid use and/or cognitive status at a single time point.

Two qualitative studies [17, 18] have examined the reverse path, with results suggesting that indeed people with cognitive decline encounter barriers to hearing-aid use that are relatable to memory and cognitive impairment. However, these studies also lack control for the other (in this case, forward) path.

In this paper, we attempt to separately estimate the magnitude of the effects of the two potential causal paths. Specifically, using data from Veterans Affairs (VA) electronic health records (EHR) of Veterans who had received hearing aids through the VA system, we test the following hypotheses:

Hypothesis 1: Persistent hearing-aid use in cognitively intact Veterans aged 60+ years will be longitudinally associated with reduced risk of incident dementia,

Hypothesis 2: Pre-existing dementia in Veterans aged 60+ years will be longitudinally associated with reduced hearing-aid use persistence.

## Materials and methods

This work was approved by the Institutional Review Board and the Research and Development Committee of the VA Portland Health Care System (Study #03566), Data Access Request Tracker (tracking number 2014-11-066-D-A04) and VA Patient Care Services.

### Population

Data were obtained from the VA EHR system. Within the VA healthcare system, eligible Veterans are provided with hearing aids and batteries (as needed on request) free of charge, each of which are documented in the EHR system. As described previously [19], the background dataset comprised all patients in the VA EHR system with a hearing-aid order between 1 April 2012 and 31 October 2014 (*N* = 731,213). The following data were available: all diagnostic (International Classification of Diseases; ICD) and procedural codes for the period 1 January 2007 (or earliest occurrence thereafter) until 31 December 2017; hearing-aid order data between 1 April 2012 and 31 October 2014; and hearing-aid battery order data for 1 April 2012 to 31 December 2017.

Patients meeting the following criteria were included: (i) surviving until 31 December 2017; (ii) a single hearing-aid order and hearing-aid fitting, between 1 April 2012 and 31 October 2014, with at most 180 days between order date and fitting date; (iii) ≥60 years of age at time of hearing-aid fitting and (iv) audiometric data in the EHR. After applying these criteria, the initial sample size was *n* = 380,794. Of these, 98.9% were male.

**Figure 1:**
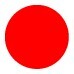
Flowcharts showing the criteria used to determine patient inclusion and resulting Ns for analysis 1 (left) and analysis 2 (right). See text for further explanation.

This initial sample was filtered by additional temporal and diagnostic criteria to extract subgroups of patients for the specific purposes of testing the above hypotheses 1 and 2 in analyses 1 and 2, respectively (see Figure 1).

### Analysis 1: Does persistent hearing-aid use reduce the risk of incident dementia?

#### Sample

The purpose of this analysis is to examine the forward path (hearing-aid use → cognitive function), corresponding to hypothesis 1. The simplest approach would be to model the likelihood of incident dementia in a specified time interval post hearing-aid fitting in those who were dementia-free at the time of hearing-aid fitting. However, since the absence of a dementia diagnosis does not equate to the absence of cognitive impairment, and because hypothesis 2 states that cognitive impairment may itself result in discontinued use of hearing aids, we took precautions to insulate this analysis from effects of the reverse path (cognitive function → hearing-aid use). This was done by including only patients free of diagnoses of either dementia (see Appendix 1 for ICD-9 and ICD-10 codesets) or mild cognitive impairment (MCI; see Appendix 2 for ICD-9 and ICD-10 codesets) up to the start of the time window for monitoring incident dementia. In this way, we minimise inclusion of patients who were potentially ‘close’ to a dementia diagnosis, rendering a maximally cognitively healthy sample at the time of hearing-aid fitting.

The analysis is complicated by the ICD system switch from version 9 to 10 on 1 October 2015. In order that all monitoring of incident dementia was based on only one system, we constrained the incidence time window to begin no earlier than 1 October 2015. This is 3.5 years after hearing-aid fitting for the earliest-fitted patients in our dataset, hence 3.5 years post-fitting was chosen as the start of the incidence time window for all patients. To maintain a reasonable balance between the lengths of the hearing-aid use persistence (and prevalent conditions) window, the length of the incident dementia window and the available *N*, the patient sample for analysis 1 was restricted to those with hearing-aid fittings up to 31 December 2012. Applying these criteria, the sample size for this analysis was *n* = 72,180.

#### Variables and analysis

Incident dementia: Incident dementia was recorded if at least one diagnosis from the ICD-10 codeset (Appendix 1) occurred between 3.5 and 5 years after hearing-aid fitting.

Covariates: Binary variables were constructed for the presence of each of the following dementia risk factors, based on diagnoses recorded up to 3.5 years post hearing-aid fitting: obesity, stroke, diabetes, depression, bipolar disorder and hypertension. See Appendix 3 for the ICD-9 and ICD-10 codesets used. Additional variables included age (years) at time of hearing-aid fitting and pure-tone average hearing threshold (dB HL) computed by averaging thresholds at 0.5, 1, 2 and 4 kHz across both ears.

A binary proxy variable indicating whether hearing-aid use remained persistent at 3.5 years post hearing-aid fitting was determined for each patient based on their history of hearing-aid battery orders, according to our previously published derivation [20]. In brief, a hearing-aid user is deemed to remain persistent at time *t* if they have ordered a pack of batteries within the 18-month period leading up to *t*. VA provides enough batteries in each pack for 6 months of full-time use; hence, ‘persistence’ corresponds roughly to continued use at several hours per day.

Logistic regression was used to model the likelihood of incident dementia 3.5 to 5 years post hearing-aid fitting. The analysis controlled for hearing-aid use persistence at 3.5 years post fitting (binary), age (to second order) and the covariates listed above.

### Analysis 2: Does cognitive function at time of hearing-aid fitting predict subsequent hearing-aid use persistence?

#### Sample

The purpose of this analysis is to examine the reverse path (cognitive function → hearing-aid use), corresponding to hypothesis 2. Since hypothesis 1 states that hearing-aid use itself may affect cognitive status, we took precautions to insulate this analysis from effects of the forward path (hearing-aid use → cognitive function). This was done by including only those patients whose cognitive status (as indexed by diagnostic codes) placed them at the high or low end of the spectrum, and whose status remained unchanged from hearing-aid fitting onwards. The sample therefore comprised

- a ‘prevalent dementia’ group (*N* = 5,838) of patients whose EHRs included at least one diagnosis of dementia (ICD-9 codeset, Appendix 1) prior to hearing-aid fitting, and
- a ‘high functioning’ group (*N* = 266,910) of patients whose EHRs contained no diagnoses of dementia or MCI (ICD-9 or ICD-10 codesets, Appendices 1 and 2) whatsoever, up to 31 December 2017.

For both groups, a clearance period criterion was applied, such that patients were only included if their first recorded VA outpatient visit was at least 2 years before hearing-aid fitting.

#### Variables and analysis

Logistic regression was used to model the likelihood of hearing-aid use persistence at 3 years 2 months post-fitting. This timepoint corresponds to the time elapsing from hearing-aid fitting to the end of the data availability for the latest-fitted patients. It maximises the available time window while also equating the time windows for all patients.

The model included group (prevalent dementia versus high functioning), and additional variables known to be strongly associated with hearing-aid use persistence [19]: Multimorbidity index using the Chronic Condition Indicator [21] for the 12-month period prior to hearing-aid order, after removal of codes for hearing loss and mental health to avoid overlap with primary variables; age (years) at time of hearing-aid fitting (to second order); new hearing-aid recipient versus experienced user (binary variable provided in the EHR [19]; pure-tone average hearing threshold (dB HL).

For both analyses, the raw data and adjusted model estimates of the primary outcome were plotted by the predictor of interest and by patient age group (decade) to facilitate interpretation.

## Results

### Analysis 1: Does persistent hearing-aid use reduce the risk of incident dementia?

**Figure 2:**
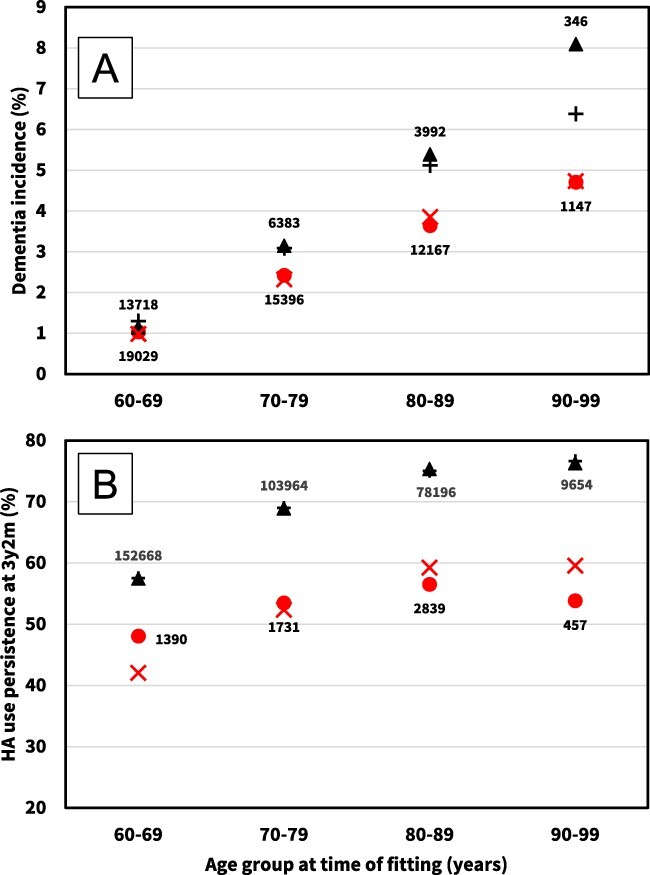
Visualisation of raw data (filled symbols) and adjusted regression models (stroke symbols) for analyses 1 and 2. Numbers indicate *N* for each data point. Patients aged 100+ not shown (*N* < 20). (A) Dementia incidence at 3.5 to 5 years post hearing-aid fitting for 72,180 patients free of dementia and MCI prior to 3.5 years post hearing-aid fitting. Adjusted model datapoints were obtained by assigning each patient their probability of incident dementia predicted by the model, then averaging in each patient age group.  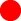 and  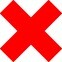, persistent;  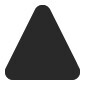 and  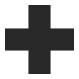, non-persistent hearing-aid users at 3.5 years post fitting. (B): Hearing-aid use persistence at 3 years 2 months post hearing-aid fitting for 272,748 patients. Adjusted model datapoints were obtained by assigning each patient their probability of hearing-aid use persistence predicted by the model, then averaging in each patient age group.  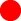 and  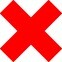, prevalent-dementia group (prevalent dementia at time of hearing-aid fitting);  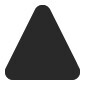 and  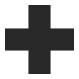, high-functioning group (no dementia or MCI at any time up to 31 December 2017).

Table 2 presents the OR, corresponding 95% CI and *P*-values for predictors in the logistic regression model of analysis 1. Figure 2A shows the unadjusted and adjusted dementia incidence estimates, grouped by hearing-aid use persistence and age decade.

As shown in Figure 2A, hearing-aid use persistence is associated with a reduced incidence of dementia, although the effect is marginal for patients below age 70 years. After full adjustment, the OR for incident dementia between 3.5 and 5 years post hearing-aid fitting is 0.73 (95% CI: 0.66–0.81) for patients who are persistent hearing-aid users at 3.5 years, relative to those who are non-persistent (Table 2).

### Analysis 2: Does cognitive function at time of hearing-aid fitting predict subsequent hearing-aid use persistence?

Table 3 presents the OR, corresponding 95% CI and *P*-values for predictors in the logistic regression model of analysis 2. Figure 2B shows the unadjusted and adjusted mean hearing-aid use persistence estimates, grouped by cognitive status and age decade.

As shown in Figure 2B, hearing-aid use persistence is about 15 percentage points higher in the high-functioning group compared to the prevalent-dementia group, regardless of age decade. After full adjustment, the OR for hearing-aid use persistence at 3 years 2 months is 0.46 (95% CI: 0.43–0.48) for patients with prevalent dementia at the time of hearing-aid fitting, relative to high-functioning patients (no dementia or MCI diagnosis up to 31 December 2017) (Table 3).

**Table 1:** Patient group characteristics at time of hearing-aid fitting. For analysis 2, we show results of comparisons of the characteristics of the high-functioning versus prevalent dementia groups using two-sided t-tests for continuous variables, and chi-square tests for percentages

**Variable**	**Analysis 1**	**Analysis 2**		
	Mean (s.d.) or %	Mean (s.d.) or %	Mean (s.d.) or %	*P*-value
Group		High functioning	Prevalent dementia	
*N*	72,180	344,500	6,418	N/A
% Male	98.9	98.5	98.8	0.014
Age (years)	73.0 (8.4)	73.6 (8.4)	78.5 (8.6)	<0.001
PTA (dB HL)	48.6 (14.4)	48.9 (14.6)	54.0 (14.8)	<0.001
Obesity (%)	33.2	N/A	N/A	
Stroke (%)	14.1	N/A	N/A	
Diabetes (%)	35.3	N/A	N/A	
Depression (%)	11.9	N/A	N/A	
Bipolar (%)	1.61	N/A	N/A	
Hypertension (%)	78.3	N/A	N/A	
% New hearing-aid recipient	N/A	44.2	31.4	<0.001
Chronic Condition Indicator	N/A	3.38 (1.89)	4.74 (2.03)	<0.001

## Discussion

The results of this study corroborate previous evidence supportive of both hypothesis 1 and 2, taking an approach which attempts to avoid some of the shortcomings of earlier studies. In particular, we have taken steps to minimise contamination between the hypothesised forward and reverse causal paths and have examined both pathways using the same dataset.

Consistent with previous findings, we found that patients over 60 years of age without cognitive impairment at the time of hearing-aid fitting, who remained persistent hearing-aid users, had 27% reduced odds of receiving a dementia diagnosis 3.5–5 years after hearing-aid fitting than patients who did not persist in hearing-aid use. Our OR of 0.73 (CI 0.66–0.81) is broadly in line with the hazard ratio of 0.82 (CI 0.76–0.89) found by Mahmoudi *et al*. [11] in a large sample of adults over 66 years of age with diagnosed hearing loss. This suggests that of the four possible mechanisms linking hearing loss and dementia described in the Introduction [3], the first (common pathology) is not dominant, since hearing-aid treatment cannot affect that pathology. Further probing of candidate mechanisms would at the very least require data on duration of hearing loss, which was not available in our dataset.

This study provides (to the authors’ knowledge) the first quantitative evidence that the diagnosis of dementia is associated with subsequent lower persistence of hearing-aid use. Patients over 60 years of age with a dementia diagnosis prior to hearing-aid fitting had 54% reduced odds of being persistent hearing-aid users (corresponding to approximately 15 percentage points in each age stratum) compared to those remaining free of MCI and dementia throughout the study time window. This may be due to reduced abilities to perform instrumental activities [4], or diverse other mechanisms, including memory problems, reduced motivation to engage in social interaction, and carers prioritising other aspects of care. Our data do not support any distinction between mechanisms on this question. Insofar as competent execution of activities of daily living is a diagnostic marker of normal cognition, persistent hearing-aid use could also be considered for inclusion. However, it would be a rather weak marker, since many people are non-persistent for reasons other than cognitive impairment [22].

The two patient groups in analysis 2 differed on several baseline characteristics (Table 1). The prevalent-dementia group showed higher age, greater hearing loss, lower proportion of new users and higher Chronic Condition Indicator. The first three of these would bias towards greater hearing-aid use persistence [19, 23] (i.e. against hypothesis 2). The fourth would bias in the opposite direction [19], but by two percentage points at most. Hence we do not consider these baseline differences problematic for the results.

**Table 2:** Logistic regression model for analysis 1; likelihood of dementia diagnosis between 3.5 and 5 years after hearing-aid fitting in patients free of dementia and MCI up to 3.5 years

**Predictor**	**OR (95% CI)**	***P* value**
Age (second-order fit; OR relative to age 75 years)		
60 years	0.19 (0.15, 0.24)	<0.001
70 years	0.64 (0.61, 0.67)	<0.001
80 years	1.42 (1.37, 1.47)	<0.001
90 years	2.09 (1.78, 2.45)	<0.001
PTA (per 10 dB increase)	1.08 (1.04, 1.12)	<0.001
Persistent hearing-aid use at 3.5 years (versus non-persistent)	0.73 (0.66, 0.81)	<0.001
Obesity (versus absence)	0.87 (0.77, 0.98)	0.02
Stroke (versus absence)	1.94 (1.73, 2.17)	<0.001
Diabetes (versus absence)	1.27 (1.14, 1.41)	<0.001
Depression (versus absence)	2.23 (1.94, 2.57)	<0.001
Bipolar (versus absence)	2.27 (1.67, 3.08)	<0.001
Hypertension (versus absence)	1.23 (1.07, 1.42)	<0.01

PTA, pure-tone average hearing threshold (dB hearing level); dB, decibel.

**Table 3:** Logistic regression model for analysis 2; likelihood of being a persistent hearing-aid user at 3 years 2 months after hearing-aid fitting

**Predictor**	**OR (95% CI)**	***P*-value**
Age (second-order fit; OR relative to age 75 years)		
60 years	0.54 (0.52, 0.56)	<0.001
70 years	0.87 (0.86, 0.88)	<0.001
80 years	1.08 (1.07, 1.08)	<0.001
90 years	1.02 (0.99, 1.06)	0.19
PTA (per 10 dB increase)	1.29 (1.28, 1.30)	<0.001
Prevalent-dementia group (versus high-functioning group)	0.46 (0.43, 0.48)	<0.001
New hearing-aid recipient (versus experienced user)	0.47 (0.46, 0.48)	<0.001
Multimorbidity (per additional body system)	0.94 (0.93, 0.94)	<0.001

PTA, pure-tone average hearing threshold (dB hearing level); dB, decibel.

### Strengths and limitations

The strengths of this study include a large longitudinal sample, key hearing data and access to information on a wide range of relevant covariates. There are also some limitations.

Our efforts to minimise contamination between the hypothesised causal paths are necessarily imperfect, and demand scrutiny. We first consider the estimation of the reverse path (analysis 2), where the contamination risks differ for the two patient subgroups. For the prevalent-dementia group, contamination from the forward path would have to take the form of persistent hearing-aid use lifting some patients out of dementia. This requires that persistent hearing-aid use reverses the slope of cognitive function over time, which seems highly unlikely. For the high-functioning group, the possible contamination would be that some patients remain high functioning only because they persist with hearing-aid use, i.e. a flattening of the slope of cognitive function versus time. In order to have any noticeable influence on the outcome of analysis 2, the flattening effect would have to be much larger than has been estimated in previous research [10]. Thus, we conclude that analysis 2 is essentially unaffected by contamination between the causal paths. Turning to contamination of the estimated forward path effect (analysis 1) arising from the reverse path, the primary risk is that undiagnosed early cognitive decline could affect hearing-aid use persistence. However, analysis 2 shows that even with fully developed dementia prior to hearing-aid fitting, hearing-aid use persistence remains at levels between 50 and 60%, corresponding to a drop of about 15 percentage points relative to high-functioning patients. Thus, it seems unlikely that any effect of undiagnosed early cognitive decline on hearing-aid use persistence could be strong enough to render the results of analysis 1 a substantial misrepresentation of the effect of the forward path. It remains the case that a stringent demonstration of causality would require a different study design.

The time window for dementia incidence in analysis 1 was only 18 months. While this is short in comparison to typical rates of cognitive decline (and thus less reliable for detecting incidence), this weakness is at least to some extent compensated by the relatively large sample size.

The Veteran population is not representative of the general population with respect to health, demographic status or psychosocial characteristics [24]. Hence, caution is needed when considering the generalisability of our findings. Nevertheless, there is no reason to think that the underlying mechanisms linking hearing loss, hearing-aid use and dementia would be different in a non-Veteran population.

One might contend that the care processes in the VA Healthcare System only represent one approach amongst many, and that the effects seen in analysis 2 are dependent on specific aspects of the VA system. While there is theoretical merit to this argument, VA care processes vary considerably across a wide network of locations and settings, and are not overly standardised. Furthermore, previous work with the same patient sample [19] indicates that on average, self-reported outcomes from hearing-aid fittings in the VA system are at least as good as those in other healthcare systems.

Due to the nature of our dataset, we had to evaluate hearing-aid use persistence at slightly different timepoints in the two analyses, introducing an arbitrary bias. However, analyses on a superset of the present dataset show that hearing-aid use persistence is very stable from about 2 years after hearing-aid fitting onwards [20].

While the large, longitudinal sample from an EHR is a strength of this study, utilising EHR data for research has some limitations. Covariate selection was limited to variables for which data were available and reliable. For example, analyses did not adjust for socioeconomic status (e.g. education) and history of smoking, both of which are risk factors for dementia [1] and may be related to hearing-aid use [25–27]. Similarly, because potential covariates were limited to measures derived from diagnostic and/or procedural codes, these analyses did not adjust for social isolation [2, 25] or psychosocial constructs (e.g. locus of control, self-efficacy, motivation) that may be associated with both dementia and hearing-aid use given their associations with healthy ageing and healthcare utilisation [28,29]. Further, relying on diagnostic codes rather than research-grade diagnostic procedures to establish health-state variables can introduce biases due to variations in coding practice and late- or undiagnosed conditions, but is unavoidable when harvesting insights from routine clinical data.

Finally, by requiring survival to 31 December 2017, we could also have introduced some distortion of the observed effects, since dementia is independently associated with excess mortality [30]. However, since the dementia- and MCI-free group for analysis 2 was identified on the basis of survival to the end of the study period, not including a survival criterion for other groups would itself have introduced bias.

## Conclusions

This is the first study to combine longitudinal data regarding dementia diagnoses with continuous assessment of hearing-aid use in a large patient sample. The results are consistent with the hypothesis that untreated hearing problems increase risk of dementia and considering the potential underlying neurological mechanisms it would be surprising if we had found otherwise. Moreover, and not unexpectedly, cognitive impairment appears to be a factor in the discontinuation of hearing-aid use. This implies that lack of or inconsistent hearing-aid use could promote cognitive decline both in those with, and those without, dementia, raising the prospect of a vicious cycle of untreated hearing problems and cognitive decline. Future research studies investigating this must be designed in such a way as to account for the effects of cognitive decline itself on hearing-aid use persistence. In a clinical context, any protective effect of hearing-aid use against dementia will not be adequately achieved unless devices and care processes are usable by and accessible to the target population. To encourage and promote persistent hearing-aid use, more dementia-friendly devices and care processes are needed.

## Supplementary Material

aa-22-0569-File002_afac266
