# Supplementary material for: Dementia and hearing-aid use: a two-way street
Source: Age Ageing. 2022 Dec 19;51(12):afac266. doi: 10.1093/ageing/afac266 (PMC9792081; doi:10.1093/ageing/afac266)
Supplement: aa-22-0569-File002_afac266 [file aa-22-0569-file002_afac266.pdf]

Dementia and hearing-aid use: a two-way street

Supplementary material

Appendix 1: Codesets for dementia, ICD-9 and ICD-10.

For diagnosis epochs up to 2015-09-30, the following ICD-9 codes were deemed indicative of dementia primarily associated with age-related endogenous causes:

- 290.XX (dementias)
- 294.1X (dementia in conditions classified elsewhere), 294.2X (dementia, unspecified)
- 331.0 (Alzheimer's disease), 331.1X (frontotemporal dementia), 331.82 (dementia with Lewy bodies).

For diagnosis epochs from 2015-10-01 onwards, ICD-10 was operative. Domain knowledge and standard translation tools were used to arrive at the following ICD-10 codes, deemed to provide the closest available match to the above ICD-9 set of codes:

- F01.XX (vascular dementia)
- F02.XX (dementia in other diseases classified elsewhere)
- F03.XX (unspecified dementia)
- G30.X (Alzheimer's disease)
- G31.0X (frontotemporal dementia)
- G31.1 (senile degeneration of brain, not elsewhere classified)
- G31.83 (dementia with Lewy bodies).

Appendix 2: Codesets for Mild Cognitive Impairment (MCI), ICD-9 and ICD-10.

For diagnosis epochs up to 2015-09-30, the following ICD-9 codes was deemed indicative of MCI:

- 331.83 (mild cognitive impairment, so stated)

For diagnosis epochs from 2015-10-01 onwards, the following ICD-10 codes were deemed indicative of MCI:

- G31.84 (mild cognitive impairment, so stated)
- R41.81 (age-related cognitive decline).

Appendix 3: Codesets for dementia risk factors, ICD-9 and ICD-10.

For diagnosis epochs up to 2015-09-30, the following ICD-9 codes were deemed indicative of the given risk factor:

- Obesity: 278.0X (overweight and obesity), 278.1 (localized adiposity).
- Stroke: All codes within 430-438 (cerebrovascular disease).
- Diabetes: All codes within 249 (secondary diabetes mellitus) and 250 (diabetes mellitus).
- Depression: 296.2X (major depressive disorder single episode), 296.3X (major depressive disorder recurrent episode), 296.82 (atypical depressive disorder).
- Bipolar disorder: 296.5X (bipolar I disorder, most recent episode (or current) depressed), 296.6X (bipolar I disorder, most recent episode (or current) depressed), 296.7 (bipolar I disorder, most recent episode (or current) unspecified), 296.8X (other and unspecified bipolar disorders) except for 296.82.
- Hypertension: All codes within 401 (essential hypertension) and 405 (secondary hypertension).

For diagnosis epochs from 2015-10-01 onwards, the following ICD-10 codes were deemed indicative of the given risk factor:

- Obesity: E65 (localized adiposity), E66.XX (overweight and obesity).
- Stroke: All codes in sections I60-I69 (cerebrovascular diseases).
- Diabetes: All codes in sections E08-E13 (diabetes mellitus).
- Depression: All codes in F32 (major depressive disorder, single episode) and F33 (major depressive disorder, recurrent).
- Bipolar disorder: All codes in F31 (bipolar disorder).
- Hypertension: All codes in I10 (essential (primary) hypertension), I15 (secondary hypertension), I16 (hypertensive crisis).

[END]
